# Establishment of immortalized mesenchymal stem cells derived from the submandibular glands of tdTomato transgenic mice

**DOI:** 10.3892/etm.2015.2700

**Published:** 2015-08-24

**Authors:** SHINJI FURUKAWA, YUKINORI KUWAJIMA, NAOYUKI CHOSA, KAZURO SATOH, MASATO OHTSUKA, HIROMI MIURA, MINORU KIMURA, HIDETOSHI INOKO, AKIRA ISHISAKI, AKIRA FUJIMURA, HIROYUKI MIURA

**Affiliations:** 1 Division of Orthodontics, Department of Developmental Oral Health Science, Iwate Medical University School of Dentistry, Morioka, Iwate 020-8505, Japan; 2 Division of Cellular Biosignal Sciences, Department of Biochemistry, Iwate Medical University, Yahaba, Iwate 028-3694, Japan; 3 Department of Molecular Life Science, Division of Basic Medical Science and Molecular Medicine, Tokai University School of Medicine, Isehara, Kanagawa 259-1193, Japan; 4 Division of Functional Morphology, Department of Anatomy, Iwate Medical University, Yahaba, Iwate 028-3694, Japan

**Keywords:** tdTomato transgenic mice, submandibular gland, mesenchymal stem cells, osteogenic differentiation, adipogenic differentiation

## Abstract

Transgenic mice that overexpress the red fluorescent protein tdTomato (tdTomato mice) are well suited for use in regenerative medicine studies. Cultured cells from this murine model exhibit strong red fluorescence, enabling real-time *in vivo* imaging through the body surface of grafted animals. Mesenchymal stem cells (MSCs) have marked potential for use in cell therapy and regenerative medicine; however, the mechanisms that regulate their dynamics *in vivo* are poorly understood. In the present study, an MSC line was derived from the submandibular gland fibroblasts of tdTomato mice. The fluorescent signal from this cell line was observed in organs throughout the body, as well as in salivary glands. Primary culture cells derived from the submandibular gland were immortalized with SV40 large T antigen (GManSV cells); these cells exhibited increased migratory ability, as compared with those isolated from the sublingual gland. GManSV cells were tdTomato-positive and exhibited spindle-shaped fibroblastic morphology; they also robustly expressed mouse MSC markers: Stem cell antigen-1 (Sca-1), CD44, and CD90. This cell line retained multipotent stem cell characteristics, as evidenced by its ability to differentiate into both osteogenic and adipogenic lineages. These results indicate that Sca-1^+^/CD44^+^/CD90^+^-GManSV cells may be useful for kinetic studies of submandibular gland-derived MSCs in the context of *in vitro* co-culture with other types of salivary gland-derived cells. These cells may also be used for *in vivo* imaging studies, in order to identify novel cell therapy and regenerative medicine for the treatment of salivary gland diseases.

## Introduction

Saliva, which is produced by the salivary glands, consists of mucus, various electrolytes, glycoproteins, enzymes, and antibacterial compounds. Therefore, dysfunction or disruption of saliva production presents a significant clinical concern. In particular, hyposalivation, which is a characteristic of xerostomia, may significantly reduce the quality of life of patients (1,2). Hyposalivation is most common in patients with Sjögren's Syndrome (3), ectodermal dysplasias (4–6), head and neck cancer following γ-irradiation therapy (7), or as a side effect of various medications. With regards to therapeutic strategies for the treatment of these diseases, previous studies have adopted regenerative medicine strategies using stem cell sources in order to engineer artificial salivary tissue that can mitigate the effects of xerostomia and hyposalivation (8,9). Removed via lateral parotidectomy, *in vitro* isolation and characterization of stem cells from the human parotid gland, has previously been achieved (10). Subsequent flow cytometric analysis demonstrated that these stem cells were strongly positive for the classic mesenchymal stem cell (MSC) markers: CD13, CD29, CD44, and CD90, and negative for the key hematopoietic stem cell (HSC) markers, CD34 and CD45. MSCs are multipotent stem cells capable of differentiating into numerous cell lineages, including chondrocytes, adipocytes, osteoblasts, acinar cells, and salivary epithelial cells (11–14) Therefore, MSCs have been highlighted as powerful candidates for experimental investigations (*in vitro* and *in vivo*) and clinical treatment due to their anti-inflammatory effects, low immunogenicity and potential to repair damaged tissues (11–15). In this regard, the salivary gland-derived stem cells exhibit MSC-like characteristics, as they can be differentiated into adipogenic, osteogenic, and chondrogenic lineages. Therefore, MSCs may exert useful effects for the regeneration and functional restoration of the salivary gland.

Fluorescent transgenic (Tg) mice have been extensively used for analyses of gene function, cellular dynamics, and bioimaging. In addition, Tg mice have been used as *in vivo* models for the study of numerous diseases (16). Fluorescent proteins (FPs) span the entire color spectrum, and may be used to color-code cells of a specific genotype or phenotype. For example, the behavior of one cell type labeled with green FP (GFP) can be compared with another cell type labeled with red FP (RFP) *in vivo*. Alternatively, host and donor cells can be differentially labeled with FPs; in this way, a Tg mouse constitutively expressing GFP can be the recipient of transplanted cells that express RFP, in order to visualize the interaction between the two cell types in real time. The current FP color palette includes modified proteins based on *Aequorea* GFP, as well as various FPs that have been cloned from other marine organisms and improved for live-cell imaging applications via genetic engineering (17,18). The mushroom anemone *Discosoma striata* was the source of an RFP known as DsRed (19); subsequently, a much brighter dimeric RFP, called tdTomato, was generated (20). This probe is useful for applications that require minimal exposure to excitation illumination to maintain cell viability. The authors of the present study previously generated a series of fluorescent Tg mouse lines using the pronuclear injection-based targeted transgenesis (PITT) method, and demonstrated that these mice exhibit a high level of transgene expression (21). In addition, unlike those developed by random-integration-based transgenesis, the *Rosa26*^CAG::FP^ mice generated by the PITT method exhibited stable, reproducible and uniform FP expression in various organs, including the liver, kidney and intestine, indicating the potential of these mice for use in chimeric and transplantation analyses (22).

The present study established an MSC line, GManSV, using submandibular gland (Gman) fibroblast like-cells from *Rosa26*^CAG::tdTomato^ Tg mice (tdTomato mice), which were immortalized by transfection with a plasmid expressing SV40 large T antigen. The aim was to evaluate the applicability of GManSV cells for use in kinetic studies of MSC *in vitro* and *in vivo*.

## Materials and methods

### 

#### Rosa26^CAG::tdTomato^ Tg mice (tdTomato mice)

The study was approved by the ethics committee of the Animal Studies Committee at Iwate Medical University (nos. 21-039 and 23-075; Center for In Vivo Science, Iwate Medical University, Yahaba, Iwate, Japan). tdTomato mice were maintained by crossbreeding homozygous mutant mice. C57BL/6 wild-type and tdTomato mice were maintained at the Iwate Medical University Center for In Vivo Science under standard conditions (23°C; 12-h light/dark cycle) with sawdust bedding and food (CE-2; CLEA Japan, Inc., Tokyo, Japan) and water *ad libitum*. The tdTomato mice were generated using the PITT method and the red fluorescence of tdTomato was observed in all organs of the transgenic mice, as described in a previous study (21).

#### Preparation and observation of tissue sections

The tdTomato mice were sacrificed by excessive inhalation of 90–100% CO2. Whole mice were subsequently cryo-embedded in 5% carboxymethyl cellulose in hexane cooled by liquid nitrogen without fixation and decalcification. The samples were placed in a CM3050S cryostat (cutting edge angle: 7–10°, CT: −22°C, OT: −22°C; Leica Microsystems GmbH, Wetzlar, Germany) and 10-µm frontal serial cryosections were cut using a TC-65 tungsten carbide blade (Leica Microsystems GmbH) according to Kawamoto's film-transfer method (23,24) with Cryofilm TYPE I-B (Leica Microsystems GmbH). Neighboring serial sections were counterstained with hematoxylin-eosin (Wako Pure Chemical Industries, Inc., Osaka, Japan). The red fluorescence of tdTomato in the prepared sections was observed using a VIOREVO BZ-9000 fluorescence microscope (Keyence Corporation, Osaka, Japan) with a Texas Red fluorescence filter.

#### Isolation of primary culture cells from salivary glands

After the capsula covering the salivary gland of 1-week-old tdTomato mice was removed, the gland body was extracted. The GMan and sublingual gland (GLin) were segmented as a tissue mass using scissors. The glands were allowed to attach to the bottom of a 35 mm plastic cell culture dish, and the cells were cultured in Dulbecco's modified Eagle's medium (DMEM; Sigma-Aldrich, St. Louis, MO, USA) supplemented with 10% fetal bovine serum (FBS; HyClone, GE Healthcare Life Sciences, Logan, UT, USA) for 1 week at 37°C in a humidified atmosphere containing 5% CO2. Adherent cells that grew out from the tissue mass were then placed in 90 mm culture dishes and cultured in DMEM supplemented with 10% FBS. Once the culture reached 80% confluence, the cells were replated.

#### Measurement of cell migration in salivary gland-derived primary cells

Single cell migration was evaluated by measuring the distance moved by the cell in a time-lapse period using a VIOREVO BZ-9000 fluorescence microscope (Keyence Corporation) with a Texas Red fluorescence filter, in addition to an IX70 phase contrast microscope (Olympus Corporation, Tokyo, Japan). Images were captured from ~75 primary culture cells derived from GLin or GMan 200 times every 10 min for up to 33 h. Two-dimensional moving distances of the cells were measured using BZ-II image analysis software (Keyence Corporation).

#### Establishment of immortalized GMan cells

The expanded cells (1.0×10^5^) derived from the GMan of tdTomato mice were transfected with a pBABE-puro-SV40LT plasmid containing a puromycin resistance gene (Addgene Inc., Cambridge, MA, USA) using Lipofectamine LTX (Invitrogen Life Technologies, Carlsbad, CA, USA) for 6 h at 37°C in 5% CO2, according to the manufacturer's instructions. The cells were exposed to DMEM supplemented with 10% FBS and 1 µg/ml puromycin (Invitrogen Life Technologies) for 12–15 days. The surviving cells were trypsinized (Invitrogen Life Technologies) and allowed to grow in 90 mm culture dishes.

#### Detection of MSC markers by flow cytometry

Following selection with puromycin, GMan-derived cells (1.0×10^5^) were suspended in phosphate-buffered saline supplemented with 0.5% FBS and 2 mM EDTA. The cells were incubated with fluorescein isothiocyanate (FITC)-conjugated anti-mouse Sca-1 (1:10; 130-102-297), anti-mouse CD44 (1:10; 130-102-511), or anti-mouse CD90 (1:10; 130-102-452) antibodies for 1 h at 4°C in the dark. All FITC-conjugated antibodies were purchased from Miltenyi Biotec GmbH (Bergisch Gladbach, Germany). Image acquisition was performed with an EPICS XL ADC system (Beckman Coulter, Inc., Brea, CA, USA).

#### Osteogenic and adipogenic differentiation

The *in vitro* differentiation method used here was reported in our previous study (25). Briefly, to investigate osteogenic differentiation, bone matrix mineralization was evaluated using 1% Alizarin Red S (Sigma-Aldrich) staining. To investigate adipogenic differentiation, lipid droplets were stained with 0.18% Oil Red O (Sigma-Aldrich).

#### Statistical analysis

The experiments were repeated at least three times and representative images or data are presented. Statistical data are presented as the mean ± standard deviation. Differences between samples were statistically analyzed using paired two-tailed Student's t-tests. P<0.05 was considered to indicate a statistically significant difference.

## Results

### 

#### Detection of tdTomato fluorescence in salivary glands

**Figure 1.**
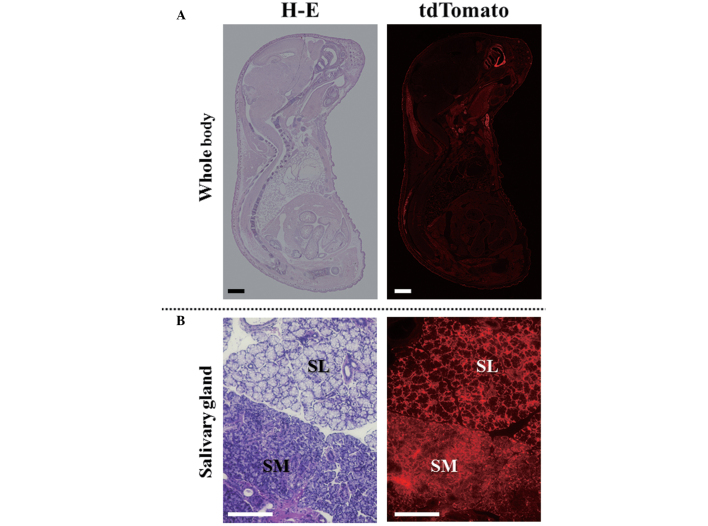
Red fluorescence of tdTomato was evaluated in (A) all organs and (B) salivary glands. Tissue sections for the whole body were prepared by the film-transfer method, as described in Materials and Methods. tdTomato was observed using a VIOREVO BZ-9000 fluorescence microscope with a Texas Red fluorescence filter. Neighboring serial sections were counterstained with hematoxylin-eosin (H-E). SM, submandibular; SL, sublingual. Scale bar, 5 mm in (A) and 500 µm in (B).

The red fluorescence of tdTomato was detected in tissue sections prepared from tdTomato mice. As shown in Fig. 1, tdTomato fluorescence was detected ubiquitously in all organs (Fig. 1A). In addition, fluorescence was detected in the cells that constitute the salivary gland tissues (Fig. 1B). There was no difference in fluorescence intensity between the GLin and GMan glands.

#### Evaluation of the migratory ability of salivary gland-derived primary cells

**Figure 2.**
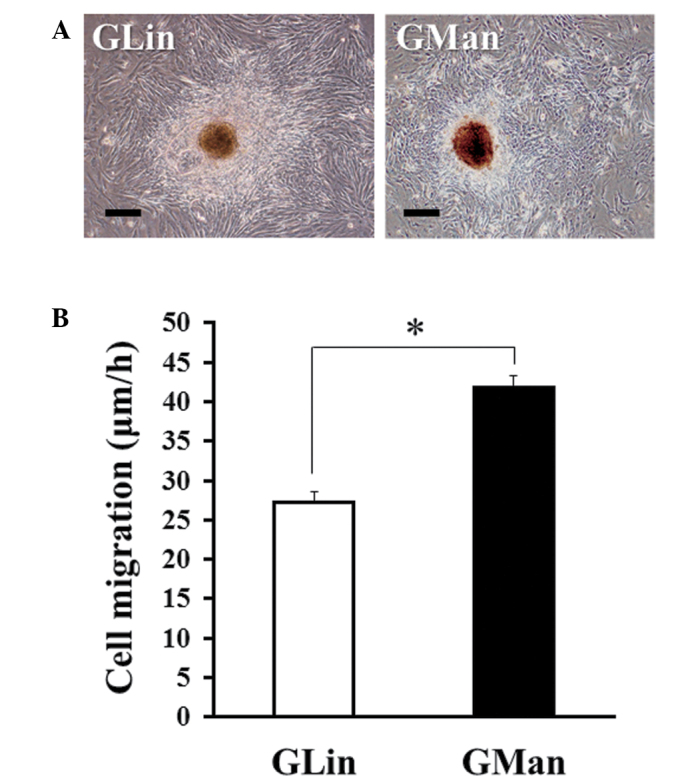
Migratory ability of primary culture cells derived from the submandibular gland (GMan) was significantly higher, as compared with the sublingual gland (GLin)-derived cells. (A) GLin and GMan of 1-week-old tdTomato mice were allowed to attach to the bottom of a 35-mm dish and cultured for 1 week. Images of the cells were captured using an phase contrast microscope. Scale bar, 200 µm. (B) Cell migratory ability was measured as described in Materials and Methods. A total of 75 primary culture cells derived from GLin or GMan were measured. Data are presented as the mean ± standard deviation. *P<0.05.

The present study subsequently isolated primary cultured GLin and GMan cells from the tdTomato mice. As shown in Fig. 2, the cells that grew out from the tissue masses possessed a fibroblast-like morphology. Notably, although the morphology of GLin and GMan cells was similar (Fig. 2A), the migratory ability of GMan cells was significantly higher, as compared with that of GLin cells (Fig. 2B). Usually, MSCs maintain a high migratory ability (26–28). Therefore, the GMan cells were selected for the establishment of the MSC line.

#### Establishment of an MSC line from GMan cells

**Figure 3.**
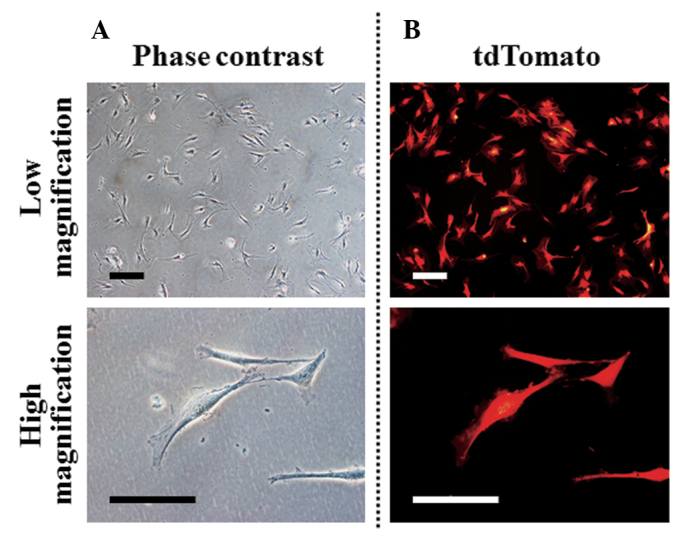
Submandibular gland cells (GMan) immortalized with SV90 large T antigen (GManSV) exhibited spindle-shaped fibroblastic morphology and expressed tdTomato. Fibroblastic primary culture cells derived from GMan of tdTomato mice were transfected with SV40 large T antigen (SV40LT) plasmid vector. Images of GManSV cells were captured with (A) a phase contrast microscope and (B) tdTomato fluorescence was detected using a microscope with a Texas Red filter. Scale bar, 200 µm in upper panel and 100 µm in lower panel.

**Figure 4.**
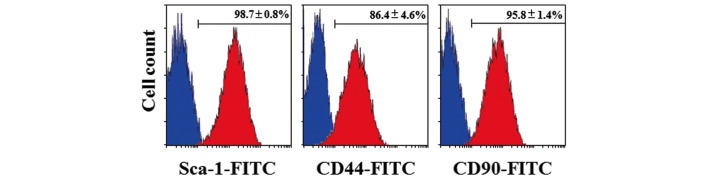
Expression of mesenchymal stem cell (MSC) markers in submandibular gland cells immortalized with SV90 large T antigen (GManSV) was analyzed by flow cytometry. Cells were incubated with fluorescein isothiocyanate (FITC)-conjugated (A) anti-mouse Sca-1, (B) anti-mouse CD44, or (C) anti-mouse CD90 antibodies for 1 h at 4°C in the dark. Image acquisition was performed using an EPICS XL ADC system. Data are presented as the means ± standard deviation.

**Figure 5.**
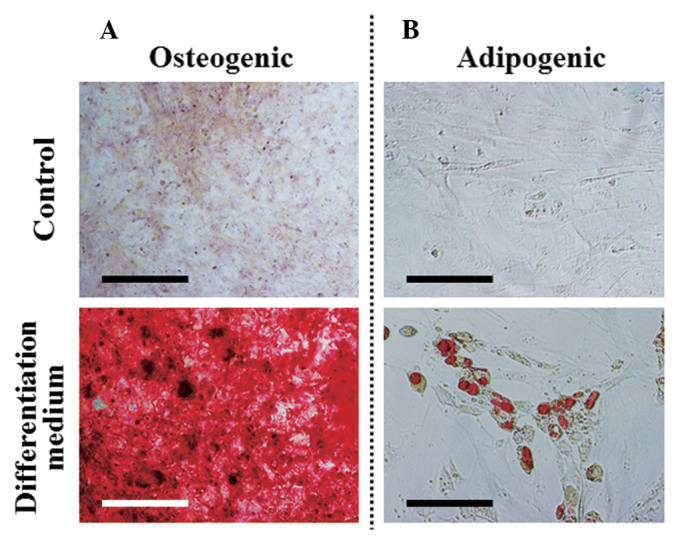
Submandibular gland cells immortalized with SV90 large T antigen (GManSV) retain multipotent stem cell characteristics and can differentiate into osteogenic and adipogenic lineages. GManSV cells were seeded onto 48-well culture plates with (differentiation medium) or without (control) (A) osteogenic or (B) adipogenic induction medium. After 2 weeks, the cells were evaluated for (A) extracellular matrix mineralization by Alizarin Red S staining and (B) for adipogenic differentiation by Oil-Red O staining. Scale bar, 100 µm.

GMan cells were immortalized using SV40 large T antigen (SV40LT) in order to produce GManSV cells. As shown in Fig. 3, these cells had a spindle-shaped fibroblastic morphology (Fig. 3A) and exhibited a strong expression of tdTomato, as determined by fluorescence imaging (Fig. 3B). Furthermore, the expression of mouse MSC markers and the differentiation potential of the cell line were determined. As shown in Fig. 4, Sca-1, one of the most functionally critical MSC markers in mice, was strongly expressed in GManSV cells (Fig. 4A). In addition, CD90 and CD44 were also highly expressed (Fig. 4B and C). In order to determine whether the cell line possessed multipotent properties, the osteogenic and adipogenic differentiation potentials of the GManSV cells were evaluated. Alizarin Red S staining demonstrated that GManSV cells could differentiate into osteoblasts (Fig. 5A). In addition, Oil Red O staining demonstrated the cells could differentiate into adipocytes (Fig. 5B). These results strongly suggest that GManSV cells retain MSC-like multipotency.

## Discussion

The present study established an MSC line from murine GMan cells overexpressing the RFP tdTomato, by immortalization of the cells with SV40LT. The SV40LT protein was selected based on its role in the infection of both permissive and non-permissive cells, leading to the production of progeny virions and malignant transformation, respectively (29,30). In addition, SV40LT has been used in cancer research for immortalization and transformation of cells (31), as primary cells are non-permissive for SV40LT, and infection with wild-type SV40LT leads to immortalization and transformation of a small percentage of infected cells. Such transformation approaches usually involve the overexpression of oncogenes and/or inactivation of tumor suppressor genes. Commonly used oncogenes include K-Ras, c-myc, cyclin-dependent kinase 4, cyclin D1, Bmi-1, and human papillomavirus 16 E6/E7, and frequently inactivated tumor suppressor genes include p53, retinoblastoma, and p16INK. In contrast to the previously mentioned oncogenes, SV40LT expression generally leads to immortalization, but not transformation (32–35). Therefore, SV40 is often used in order to avoid the excessive cellular changes that are associated with full-blown transformation.

The salivary glands of the majority of mammals consist of three main cell types: Serous-producing acinar cells, mucus-producing acinar cells, and myoepithelial cells (36). Serous-producing acinar cells possess a pyramidal morphology and join together to form spheroidal shapes; whereas mucus-producing acinar cells are cuboidal in shape and group together to form tubules. Myoepithelial cells are located near ductal openings and are associated with the contraction of ducts, in order to facilitate salivary secretion (37). Furthermore, in normal salivary glands, the mesenchymal tissue between the acinar-ductal epithelial structures (38) may also contain stem cells of mesenchymal origin, as detected in various other organs, including the bone marrow (39). Since adult stem cells are generally restricted to cell lineages of the body part of origin, previous studies have aimed to use salivary gland-derived stem cells, in order to reduce hyposalivation and restore natural function (37,40,41). Stem cells have been isolated and characterized from major salivary glands of humans and animals (42–44). The injection of these cells into the major salivary gland parenchyma has been proposed as an ideal therapeutic strategy for the restoration of irreversibly damaged gland tissue in patients with head and neck cancer who have received radiotherapy (45). To date, numerous approaches have been taken; some groups have harvested cells from the parotid gland (10) and GMan (45), whereas others have harvested cells from a combination of both glands (46), and by co-culture (47). In particular, stem cells isolated from a combination of the human parotid gland and GMan were demonstrated to partially restore salivary gland function in radiation-damaged rat salivary glands *in vivo* (46). Furthermore, previous *in vitro* studies have demonstrated that salivary gland-derived stem cells differentiate into MSC lineages, express MSC markers (CD44, CD49f, CD90, and CD105) in lieu of HSC markers (CD34 and CD45), and can differentiate into amylase-expressing cells (48–50).

Successful isolation of MSCs from various organs can be determined by a combination of criteria including morphology, surface marker phenotype, and differentiation potential (51,52). A defined panel of markers has been suggested in humans, however there are currently no standard criteria that have been proposed in mice. However, stable identification and isolation of murine MSCs using a lineage-, and Sca-1+ phenotype has been reported (53,54). CD90 may function as an activator of stem cell differentiation (55), and CD44 is a marker that is commonly detected in human MSCs (56). In the present study, the MSC line derived from GManSV cells were shown to express Sca-1, CD44, and CD90 on the cell surface. Furthermore, the GManSV cells retained the multipotent characteristics of MSCs, as they could differentiate into both osteogenic and adipogenic lineages. Therefore, these findings suggested that GManSV cells may be used as a GMan-derived MSC line for research focused on various regenerative medicine strategies.

To the best of our knowledge, the present study is the first to report a stable cell line that has been derived from tdTomato mice. The tdTomato probe is useful for applications that require minimal exposure to excitation illumination, in order to maintain cell viability. By taking advantage of this attribute, GManSV cells may be adopted as useful tools for kinetic studies of GMan-derived MSCs for *in vitro* co-culture systems with other types of salivary gland-derived cells. These cells may be well suited for *in vivo* imaging studies of cell therapy and regenerative medicine focused on salivary gland diseases.
